# Inhibition of the Phosphatidylinositol-3 Kinase Pathway Using Bimiralisib in Loss-of-Function *NOTCH1*-Mutant Head and Neck Cancer

**DOI:** 10.1093/oncolo/oyac185

**Published:** 2022-09-16

**Authors:** Faye M Johnson, Filip Janku, Mohamed A Gouda, Hai T Tran, Jitesh D Kawedia, Debora Schmitz, Hendrik Streefkerk, J Jack Lee, Clark R Andersen, Defeng Deng, Seema Rawal, Pooja A Shah, Adel K El-Naggar, Jason M Johnson, Mitchell J Frederick

**Affiliations:** Department of Thoracic/Head & Neck Medical Oncology, The University of Texas MD Anderson Cancer Center, Houston, TX, USA; The University of Texas Graduate School of Biomedical Sciences, Houston, TX, USA; Department of Investigational Cancer Therapeutics, The University of Texas MD Anderson Cancer Center, Houston, TX, USA; Department of Molecular Pathology, The University of Texas MD Anderson Cancer Center, Houston, TX, USA; Department of Thoracic/Head & Neck Medical Oncology, The University of Texas MD Anderson Cancer Center, Houston, TX, USA; Department of Thoracic/Head & Neck Medical Oncology, The University of Texas MD Anderson Cancer Center, Houston, TX, USA; Department of Pharmacy Pharmacology Research, Division of Pharmacy, The University of Texas MD Anderson Cancer Center, Houston, TX, USA; PIQUR Therapeutics AG, Basel, Switzerland; PIQUR Therapeutics AG, Basel, Switzerland; Department of Biostatistics, The University of Texas MD Anderson Cancer Center, Houston, TX, USA; Department of Biostatistics, The University of Texas MD Anderson Cancer Center, Houston, TX, USA; Department of Otolaryngology, Baylor College of Medicine, Houston, TX, USA; Department of Otolaryngology, Baylor College of Medicine, Houston, TX, USA; Department of Thoracic/Head & Neck Medical Oncology, The University of Texas MD Anderson Cancer Center, Houston, TX, USA; Department of Pathology, The University of Texas MD Anderson Cancer Center, Houston, TX, USA; Department of Neuroradiology, The University of Texas MD Anderson Cancer Center, Houston, TX, USA; Department of Otolaryngology, Baylor College of Medicine, Houston, TX, USA

**Keywords:** head and neck squamous cell carcinoma, PI3 kinase, mTOR, NOTCH1, pharmacokinetics

## Abstract

**Background:**

PI3K/mTOR inhibition leads to apoptosis of *NOTCH1*-mutant head and neck squamous cell carcinoma (HNSCC) cells. We tested the efficacy of the PI3K/mTOR inhibitor bimiralisib in patients with *NOTCH1*-mutant HNSCC.

**Methods:**

Patients with recurrent/metastatic *NOTCH1*-mutant HNSCC who had progressed during chemotherapy and immunotherapy received bimiralisib until unacceptable toxicity or progression. To assess whether *NOTCH1* mutations can be detected in blood, we measured circulating tumor DNA (ctDNA). To assess activated NOTCH1 protein levels, we quantitated cleaved *NOTCH1 (cl-NOTCH)* by immunohistochemistry.

**Results:**

Eight patients were treated, and 6 were evaluable for response. The objective response rate was 17%. For all 8 patients, median progression-free and overall survival was 5 and 7 months, respectively. Bimiralisib was well tolerated, with expected hyperglycemia. Pharmacokinetic values were consistent with published studies. *NOTCH1* mutations were detected in 83.3% of ctDNA. Staining for tumor *cl-NOTCH1* was negative. The trial closed early due to sponsor insolvency.

**Conclusion:**

Although the trial was small, outcomes with bimiralisib were better than the historical standard of care; Results will need to be confirmed in a larger trial. The lack of *cl-NOTCH1* was consistent with loss-of-function mutations and validated our mutation function algorithm. The ability to detect *NOTCH1* mutations in blood will help future studies. (ClinicalTrials.gov Identifier: NCT03740100).

## Teaser

Recurrent head and neck squamous cell carcinoma (HNSCC) remains lethal despite recent advances with immunotherapy, and effective targeted therapies are still needed. This article reports on the efficacy of the PI3K/mTOR inhibitor bimiralisib in patients with NOTCH1-mutant HNSCC.

**Lessons Learned**

- In a small cohort of patients with chemotherapy- and immunotherapy-refractory recurrent/metastatic head and neck squamous cell carcinoma (HNSCC), both the responses and the prolonged stable disease following single-agent bimiralisib appeared to be promising.
- Because *NOTCH1* loss-of-function mutations are common in other squamous cell carcinomas (esophagus, lung, and skin), these findings may translate beyond HNSCC.
- The lack of cleaved *NOTCH1* expression in tumors validated the authors’ algorithm for determining *NOTCH1* mutation function.
- The ability to detect *NOTCH1* mutations in blood will help future studies.

## Discussion

The identification of genomic alterations in HNSCC has yet to improve patient outcomes because most alterations are in tumor suppressors, including *NOTCH1,* which is mutated in ~20% of patients. Recurrent HNSCC remains lethal despite recent advances with immunotherapy, and effective targeted therapies are still needed. To address this need, we showed that PI3K/mTOR inhibition induced apoptosis in HNSCC cell lines with *NOTCH1* loss-of-function (LOF) mutations in vitro and in vivo. In a phase I study of single-agent bimiralisib, one patient with HNSCC with a *NOTCH1*-mutation was treated and experienced a partial response that lasted for 9 months. In the current study, we tested the hypothesis that the pan-class I PI3K/mTOR antagonist bimiralisib would result in cancer cell apoptosis and concomitant tumor shrinkage in *NOTCH1*-mutant HNSCC. To determine if NOTCH1 mutations can be detected in blood and monitor response to therapy, we assessed ctDNA at baseline, at week 7, and at progression. We evaluated the functional impact of NOTCH1 mutations using our novel algorithm. To determine if tumors retained any NOTCH1 protein activity, we assessed the presence of activated NOTCH1 in pretreatment tumors using an antibody that detects activated *NOTCH1 (cl-NOTCH1).*

**Figure 1.**
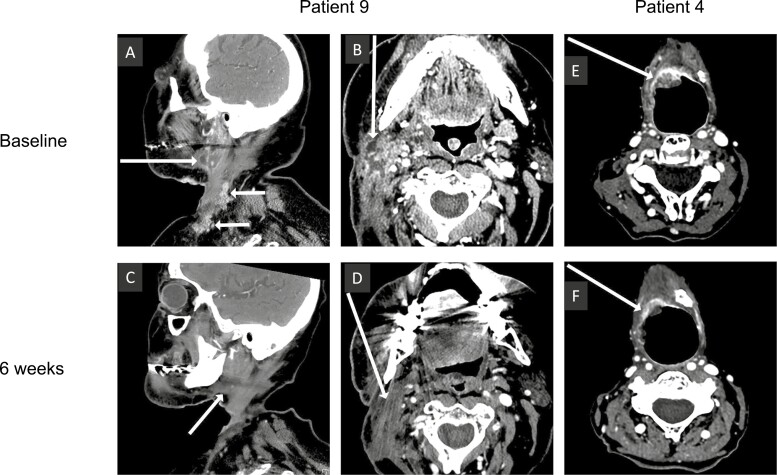
Responses to bimiralisib. Computed tomography images from patient 9 (**A**-**D**) and patient 4 (**E**, **F**) at baseline (A, B, E) and after 6 weeks of therapy (C, D, F). White arrows indicate head and neck squamous cell carcinoma tumor location.

In the 6 evaluable patients with recurrent or metastatic (R/M) HNSCC that was chemotherapy and immunotherapy resistant, bimiralisib had an objective response rate of 17%. For all 8 treated patients, the median OS and PFS was 7 and 5 months, respectively. One patient had a confirmed partial response with a 49% reduction in the target lesion size (Fig. 1A-D). She remained in the study until day 157 when the protocol was terminated. Three patients had confirmed stable disease before disease progression on days 91, 138, or 167. An additional patient was noted to have clear shrinkage on imaging. Tumor dimensions decreased from 19 mm × 16 mm to 17 mm × 8 mm on day 42 (Fig. 1E, F), but this response was technically stable disease according to RECIST. One patient had progressive disease on day 83. Two patients were not evaluable for response based on toxicity from cancer progression on day 8 and day 9.

This is the only clinical trial to test the therapeutic vulnerability of *NOTCH1*-mutant HNSCC to any class of drugs. Future studies in *NOTCH1-*mutant cancers will be facilitated by the validation of our algorithm to predict *NOTCH1* mutation function and the ability to detect *NOTCH1* mutations in blood. Although the trial was small, the outcomes were promising in a group of HNSCC patients with a very poor prognosis. Because *NOTCH1* loss-of-function mutations are common in other squamous cell carcinomas, including those of the esophagus and lung, our findings may translate beyond HNSCC.

## Trial Information

**Table:** 

Disease	Head and neck squamous cell carcinoma (HNSCC)
Stage of disease/treatment	Recurrent or metastatic
Prior therapy	Cisplatin-based chenotherapy. Anti-PD1 mmunotherapy
Type of study	Phase II, nonrandomized, non-blinded, single-arm study
Primary endpoint	The objective response rate, including confirmed complete response and confirmed partial response, according to RECIST version 1.1
Secondary endpoints	Safety, progression-free survival, overall survival, pharmacokinetic analysis, and biomarkers (ctDNA, cl-NOTCH)
Investigator’s analysis	Active and should be pursued further

## Additional Details of Endpoints or Study Design

### Patient Selection

Adults with an Eastern Cooperative Oncology Group performance status of ≤2, measurable disease according to Response Evaluation Criteria in Solid Tumors (RECIST) version 1.1,^1^ and histologically confirmed recurrent or metastatic (R/M) HNSCC for which no standard curative or life-prolonging therapy was available were eligible. All patients had already received platinum chemotherapy and α-PD1 immunotherapy unless unable to tolerate them.

The presence of a NOTCH1 mutation in the HNSCC tissue had to be determined by Clinical Laboratory Improvement Amendments–certified sequencing results. We excluded NOTCH1 splice mutations in exons 33 or 34; truncating mutations in carboxy-terminal regions associated with activation, including the transcription activation domain and proline-, glutamate-, serine-, and threonine-rich domains; and non-truncating mutations in the negative regulatory region and heterodimerization domains. Patients with NOTCH1 mutations in all other regions were further evaluated for eligibility using an algorithm as described^2^ which predicts the likelihood of inactivation based on both the specific NOTCH1 protein domain affected and the PROVEAN/SIFT^3,4^ scores of amino acid changes.

We defined adequate bone marrow, liver, and renal functions as follows: platelet count ≥100 × 10^9^/L, absolute neutrophil count ≥1.5 × 10^9^/L, hemoglobin ≥9 g/dL, alanine aminotransferase and aspartate aminotransferase ≤2.5 × upper limit of normal, serum total bilirubin ≤upper limit of normal, glomerular filtration rate ≥30 mL/minute (Cockcroft-Gault), and fasting plasma glucose ≤150 mg/dL.

### Study Design

All patients received 140 mg bimiralisib (PQR309) orally once daily on 2 consecutive days followed by 5 days without treatment weekly until unacceptable toxicity, tumor progression, patient request for withdrawal, investigator judgment, or death. The primary endpoint was to determine the objective response rate, including confirmed complete response and confirmed partial response according to RECIST version 1.1.^1^ Secondary and exploratory endpoints included safety, progression-free survival (PFS), overall survival, pharmacokinetic (PK) analysis, and biomarkers described below.

To minimize accrual if bimiralisib was ineffective, we used a Simon optimal 2-stage design. In order to have 80% power to detect a response rate of 30% (one-sided α = 0.05 and β = 0.20), we planned to enroll up to 10 patients in the first stage. If ≥2 patients had an objective response, then we had planned to enroll an additional 19 patients in the second stage. Radiologic tumor assessments were performed using computed tomography at baseline and every 6 weeks. Safety was continuously monitored.

The trial was registered on ClinicalTrials.gov (NCT03740100). All patients provided written, informed consent. The study was performed in accordance with the ethical principles of the declaration of Helsinki and in agreement with the International Council for Harmonization guidelines on Good Clinical Practice E6 R2. One patient (patient 9) transitioned to an expanded access program to receive bimiralisib when NCT03740100 closed and the patient received identical treatment and assessments as in NCT03740100 until the expanded access program closed.

### Statistical Analysis

Demographic and clinical characteristics were summarized using univariate statistics, including mean, median, and standard deviation or frequency and percentage. Objective response rate was summarized by frequency and percentage with a 2-sided 95% Agresti-Coull CI.^5^ Duration of response, PFS, and overall survival were analyzed using the Kaplan-Meier method.^6^ All statistical analyses were performed using R statistical software (version 3.6.3, R Core Team, 2020). Survival modeling was performed using the “survival” package.^7^

Time to response, duration of response, PFS, and overall survival were analyzed using the Kaplan-Meier method.^6^ Time to response was defined as the time from first study drug administration to the first documentation of response (complete or partial). Duration of response was defined as the time from the date of the first confirmed response to the first documentation of disease progression. PFS was defined as the time from first study drug administration to disease progression or death due to any cause. Overall survival was defined as the time from first study drug administration to death due to any cause.

### PK Analysis

Blood was collected before oral dose administration and 1 hour after oral dose administration on days 1, 8, 22, 43, and 64 after the start of therapy. Bimiralisib levels were determined as previously described using high-performance liquid chromatography^8-11^ by Charles River Laboratories (Ashland, Ohio). The pre-dose levels were the trough levels and post-dose levels were the assumed peak serum concentration (*C*_max_) values. *C*_max_ and trough accumulation ratios were calculated by dividing each patient’s treatment day concentration by their initial available drug concentration—day 1 for *C*_max_ and day 8 for trough. Statistical differences among groups were identified by analysis of variance using Statistica version 13.1 (TIBCO Software Inc., Palo Alto, California); *P* < .05 was considered statistically significant

### Cell-Free DNA

Peripheral blood samples were collected in K3 EDTA tubes from patients who consented to the optional blood collection protocol. Samples were processed within 30 minutes of collection. Plasma was obtained through centrifugation at 1500-2000 g for 10 minutes at 4 °C. Plasma was stored at 80 °C until further processing. Cell-free DNA was isolated using QIAamp Circulating Nucleic Acid Kit (QIAGEN, Hilden, Germany) and was initially quantified using Quant-iT Picogreen dsDNA Assay Kit (Thermofisher Scientific, Massachusetts) on SpectraMax M2 (Molecular Devices, California). Whenever feasible, 30 ng of DNA was used to prepare libraries that were sequenced on NovaSeq 6000 at The University of Texas MD Anderson Cancer Center Advanced Technology Genomics Core Laboratory using TruSight Oncology 500 ctDNA kit (TSO500; Illumina, California). TSO500 is a targeted next-generation sequencing assay that can sequence 523 genes and detect different DNA variants, including exon coverage. Bioinformatics analysis was done using DRAGEN analysis software and DRAGEN server version 3 (Illumina). We assessed the detection rate and variant allele frequency (VAF) of NOTCH1 mutations that were detected in tissue. For further analysis, the obtained mutations were filtered to exclude synonymous mutations, germline mutations, mutations that are not annotated in the Catalogue of Somatic Mutations in Cancer database, and mutations with FATHMM prediction as non-pathogenic.

### Immunohistochemistry (IHC) for NOTCH1

To assess activated NOTCH1 protein levels in archival tumor material, we quantitated cleaved NOTCH1 (cl-NOTCH1, NICD) using an anti-cl-NOTCH1 antibody that was previously validated^12-14^ using IHC. The anti-cl-NOTCH1 antibody from Cell Signaling Technology (Cat # 4147) was previously validated to work in IHC to detect cl-NOTCH1 in formalin-fixed, paraffin-embedded sections. The anti-cl-NOTCH1 antibody recognizes an epitope on the intracellular NOTCH1 fragment that becomes accessible only when NOTCH1 is cleaved between Gly1743 and Val1744 in the transmembrane domain, which happens only during NOTCH1 activation or signaling.

Formalin-fixed, paraffin-embedded slides from patients or control cell pellets were dewaxed, treated with a 3:1 ratio of EZ antigen retrieval buffer EZ AR10 (pH 10) to EZ AR2 (pH 8.5), microwaved for 10 minutes at 98 °C, rinsed in deionized H2O and phosphate-buffered saline, and loaded onto an IntelliPATH autostainer for detection with a rabbit monoclonal antibody to cl-Notch1. Briefly, slides were washed in BOND buffer (Leica Biosystems), incubated with Leica endogenous peroxide blocker for 5 minutes, rinsed, incubated for 30 minutes with anti-cl-NOTCH1 diluted 1:100 in antibody dilution buffer (Cell Signaling Technology, #12378), washed, detected with Leica BOND Polymer anti-rabbit horseradish peroxidase for 8 minutes, rinsed, and developed with BioGenex DAB (prediluted at 1 drop per milliliter) for 5 minutes before staining with hematoxylin. All autostainer incubations were at room temperature. A positive control cell line for IHC was created by first knocking out wt NOTCH1 in the HNSCC cell line FADU with CRISPR and then expressing a cDNA corresponding to the cleaved/activated intracellular NOTCH1 molecule that was driven by a doxycycline-inducible promoter. We had planned to use a semi-quantitative/ordered categorical expression scoring system for IHC.^12-14^

## Drug Information

**Table:** 

Generic/working name	Bimiralisib
Company name	PIQUR Therapeutics AG (Basel, Switzerland)
Drug type	Small molecule
Drug class	PI3K/mTOR
Dose	140
Unit	Mg
Route	Oral
Schedule of administration	Daily on 2 consecutive days weekly

## Patient Characteristics

**Table:** 

Number of patients, male	6
Number of patients, female	2
Stage	IV
Age: Median (range)	65.5 (50-71) years
Number of prior systemic therapies: median(range)	3 (2-7)
Performance status: ECOG	0: 4 1: 4 2: 0 3: 0 4: 0
Cancer types or histologic subtypes	HNSCC, Oral cavity squamous carcinoma (SCC), human papillomavirus (HPV)-negative: 4; HNSCC, Oropharynx SCC, HPV-positive, 3; HNSCC, Oropharynx SCC, HPV unknown,1.

## Primary Assessment Method

**Table:** 

Title	Objective response rate
Number of patients screened	10
Number of patients enrolled	8
Number of patients evaluable for toxicity	8
Number of patients evaluated for efficacy	6
Evaluation method	RECIST 1.1
Response assessment, CR	0 (0%)
Response assessment, PR	1 (17%)
Response assessment, SD	4 (67%)
Duration assessments, median, PFS	147 days (CI: 39-199)
Duration assessments, median, TTP	167 days (CI: 83-inf)
Duration assessments, median, OS	215 days (CI: 39-inf)
Response duration	111 days
Duration of treatment	87 days

### Outcome Notes

Between January 2019 and August 2020, 10 patients consented to receive the study treatment at MD Anderson; however, 2 of these patients died of cancer-related complications prior to treatment initiation, so 8 were treated. Per study design, all patients had recurrent or metastatic HNSCC harboring NOTCH1 mutations predicted to be loss of function (LOF). All had been treated with prior immuno- and chemotherapy with the exception of patient 4 who was unable to tolerate chemotherapy. The median number of prior systemic therapies for recurrent or metastatic HNSCC was 3. Most had also received prior definitive radiotherapy. The trial was closed early because the sponsoring company dissolved.

Overall, bimiralisib was well tolerated, with the expected toxicities (eg, hyperglycemia), cancer-related adverse events (AEs), and cancer progression accounting for all significant toxicity (Table 1). In total, 64 treatment-emergent AEs, defined as AEs that started or worsened during therapy, occurred during the study. All 8 patients experienced at least one treatment-emergent AE. No patients experienced an AE leading to dose reduction. AEs irrespective of causality occurring in ≥25% of patients included fatigue (3 patients, 37.5%), nausea (3 patients, 37.5%), vomiting (2 patients, 25.0%), hyperglycemia (3 patients, 37.5%), skin infection (2 patients, 25%), aspiration pneumonia (2 patients, 25.0%), rash (2 patients, 25.0%), and confusional state (2 patients, 25.0%).

**Table 1.** Treatment-emergent adverse events by severity.

Adverse event	Bimiralisib (*n* = 8), *n* (%)			Total number of events		
	Grade 1	Grade 2	Grade 3/ 4	Grade 1	Grade 2	Grade 3/ 4
Any adverse event	8 (100.0)	3 (37.5)	5 (62.5)	29	13	15
General disorders and administration site conditions	5 (62.5)	1 (12.5)	1 (12.5)	6	1	1
Fatigue	2 (25.0)	1 (12.5)	0	2	1	0
Asthenia	1 (12.5)	0	0	1	0	0
Chest pain	1 (12.5)	0	0	1	0	0
Disease progression	0	0	1 (12.5)	0	0	1
Gait disturbance	1 (12.5)	0	0	1	0	0
Pyrexia	1 (12.5)	0	0	1	0	0
Gastrointestinal disorders	2 (25.0)	3 (37.5)	0	2	5	0
Nausea	1 (12.5)	2 (25.0)	0	1	2	0
Vomiting	0	2 (25.0)	0	0	2	0
Diarrhea	0	1 (12.5)	0	0	1	0
Gastroesophageal reflux disease	1 (12.5)	0	0	1	0	0
Infections and infestations	0	2 (25.0)	3 (37.5)	0	3	3
Skin infection	0	1 (12.5)	1 (12.5)	0	1	1
Cellulitis	0	1 (12.5)	0	0	1	0
Osteomyelitis acute	0	0	1 (12.5)	0	0	1
Sepsis	0	0	1 (12.5)	0	0	1
Wound infection	0	1 (12.5)	0	0	1	0
Metabolism and nutrition disorders	1 (12.5)	2 (25.0)	2 (25.0)	1	3	2
Hyperglycemia	1 (12.5)	0	2 (25.0)	1	0	2
Decreased appetite	0	1 (12.5)	0	0	1	0
Dehydration	0	1 (12.5)	0	0	1	0
Hypocalcemia	0	1 (12.5)	0	0	1	0
Investigations	3 (37.5)	0	1 (12.5)	3	0	2
Alanine aminotransferase increased	0	0	1 (12.5)	0	0	1
Aspartate aminotransferase increased	0	0	1 (12.5)	0	0	1
Blood creatinine increased	1 (12.5)	0	0	1	0	0
Blood glucose increased	1 (12.5)	0	0	1	0	0
Platelet count decreased	1 (12.5)	0	0	1	0	0
Respiratory, thoracic and mediastinal disorders	1 (12.5)	0	3 (37.5)	1	0	4
Pneumonia aspiration	0	0	2 (25.0)	0	0	2
Acute respiratory distress syndrome	0	0	1 (12.5)	0	0	1
Cough	1 (12.5)	0	0	1	0	0
Laryngeal stenosis	0	0	1 (12.5)	0	0	1
Skin and subcutaneous tissue disorders	2 (25.0)	0	0	4	0	0
Rash	2 (25.0)	0	0	2	0	0
Dry skin	1 (12.5)	0	0	1	0	0
Pruritus	1 (12.5)	0	0	1	0	0
Nervous system disorders	1 (12.5)	1 (12.5)	0	2	1	0
Dysarthria	1 (12.5)	0	0	1	0	0
Presyncope	0	1 (12.5)	0	0	1	0
Seizure	1 (12.5)	0	0	1	0	0
Ear and labyrinth disorders	2 (25.0)	0	0	2	0	0
Ear pain	1 (12.5)	0	0	1	0	0
Tinnitus	1 (12.5)	0	0	1	0	0
Psychiatric disorders	2 (25.0)	0	0	2	0	0
Confusional state	2 (25.0)	0	0	2	0	0
Renal and urinary disorders	1 (12.5)	0	1 (12.5)	1	0	1
Renal injury	0	0	1 (12.5)	0	0	1
Urinary tract discomfort	1 (12.5)	0	0	1	0	0
Vascular disorders	1 (12.5)	0	1 (12.5)	1	0	1
Hemorrhage	0	0	1 (12.5)	0	0	1
Hypotension	1 (12.5)	0	0	1	0	0
Musculoskeletal and connective tissue disorders	1 (12.5)	0	0	2	0	0
Neck pain	1 (12.5)	0	0	1	0	0
Pain in jaw	1 (12.5)	0	0	1	0	0
Blood and lymphatic system disorders	0	0	1 (12.5)	0	0	1
Anemia	0	0	1 (12.5)	0	0	1
Eye disorders	1 (12.5)	0	0	1	0	0
Eye disorder	1 (12.5)	0	0	1	0	0
Injury, poisoning and procedural complications	1 (12.5)	0	0	1	0	0
Sunburn	1 (12.5)	0	0	1	0	0

Five patients (62.5%) experienced 14 severe AEs (SAEs, Common Terminology Criteria for Adverse Events grade ≥3). Two patients died during the study, one of local disease progression with associated infection and hemorrhage (patient 5) and one on day 8 of therapy from aspiration pneumonia (patient 7, details below), a common complication of recurrent HNSCC. Both events were unrelated to treatment with bimiralisib. During the on-treatment period, a further 10 nonfatal SAEs were reported that were not related to bimiralisib. Patient 3 chose to discontinue the study on day 9 after experiencing tumor-related laryngeal narrowing that required an emergency tracheostomy, making further travel to Houston problematic. Patient 9 had locally advanced disease with several cancer-related AEs during her 5 months participating in the study: aspiration pneumonia, local infection, and osteomyelitis.

Two SAEs—both hyperglycemia—were related to treatment with bimiralisib. Hyperglycemia is a well-known effect of PI3K inhibitors, including bimiralisib.^15^ One patient’s plasma glucose subsequently returned to near-normal values with medical management while the patient was participating in the study (patient 9). The other patient (patient 7) was hospitalized with grade 3 hyperglycemia that resolved, but he subsequently developed pneumonia, sepsis, respiratory distress, and acute renal failure due to aspiration from tumor-related dysphagia, which led to study discontinuation. Patient 4 discontinued participation in the study owing to grade 3 elevated liver function test results (alanine aminotransferase and aspartate aminotransferase) related to treatment with bimiralisib, and these levels normalized 28 days after therapy discontinuation. In total, 7 patients (87.5%) experienced 23 AEs (any severity) that were suspected to be related to treatment with bimiralisib.

**Figure 2.**
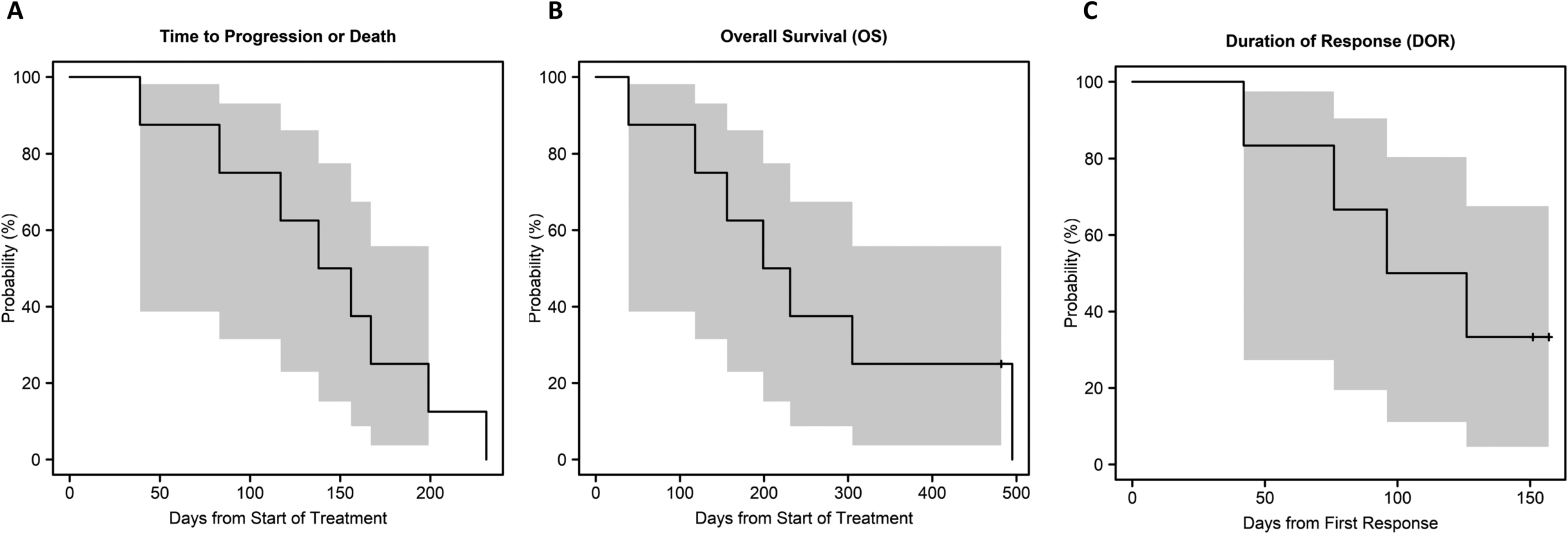
Inhibition of the phosphatidylinositol-3 kinase pathway using bimiralisib in loss-of-function NOTCH1-mutant head and neck cancer.

One patient had a confirmed partial response with a 49% reduction in the target lesion size. She remained in the study until day 157 when the protocol was terminated; her final computed tomography scan during the study showed that she maintained this response, with a 45% reduction in the target lesion size compared with baseline. Three patients had confirmed stable disease before experiencing disease progression on days 91, 138, or 167. An additional patient with stable disease according to RECIST 1.1 had a notable decrease in tumor dimensions from 19 mm × 16 mm to 17 mm × 8 mm on day 42. She discontinued therapy owing to elevated liver function test results. One patient had progressive disease on day 83. Two patients were not evaluable based on toxicity described above. Among the 6 evaluable patients, the objective response rate was 17% (1/6), with 95% CI 1-58%. For all 8 patients, the median PFS was approximately 5 months (147 days, 95% CI 39-199 days) and the median overall survival was 7 months (215 days, 95% CI 39 days-infinity). In the 6 evaluable patients, the duration of response was 111 days (Figure 2).

To assess whether NOTCH1 mutations can be detected in baseline ctDNA samples and to explore potential resistance mechanisms, we assessed ctDNA at baseline, at week 7, and at progression. Our next-generation sequencing assay covered all NOTCH1 mutations identified in patients’ tissues. Previously identified NOTCH1 mutations in tumor tissue were detected in ctDNA collected at baseline in 5 of 6 patients (83.3%) with a median VAF of 8.3% (range 0.3-26.9%). Two patients with detectable NOTCH1 had plasma samples collected at progression. Patient 1 had a similar VAF at baseline (14.7%) as at progression (12.4%). This patient had stable disease in target lesions (4% increase by RECIST) but developed a new brain metastasis, which likely did not result in increased shedding of ctDNA owing to the blood-brain barrier.^16^ Patient 2 showed an increase in VAF over time from 8.3% at baseline to 14.4% at week 7 to 56.2% at progression (24 weeks), consistent with increasing tumor burden reflected on imaging. Mutations that were detected at time of progression but not at baseline included DNMT3A P904L, PIK3CA E542K, KIT M541L, BRAF K601E, BRAF V600A, BRAF I592M, ATM A1742P, and TP53 G245D. However, there was no overlap in these novel mutations between patients 1 and 2.

Using our novel algorithm, all tumors from study patients had either truncating or missense NOTCH1 mutations predicted to be LOF.^2^ We measured NOTCH1 activity by quantitation of cl-NOTCH1 using IHC. Ligand binding to NOTCH1 exposes it to stepwise cleavage, releasing intracellular cl-NOTCH1 that translocates to the nucleus and alters gene expression. The anti-cl-NOTCH1 antibody^12^ recognizes an epitope on the intracellular NOTCH1 fragment that becomes accessible only when NOTCH1 is cleaved and does not recognize the NOTCH1 pro-form or inactive heterodimers prior to ligand activation and receptor cleavage.

We examined whether the staining patterns for cl-NOTCH1 in patient tumor biopsies were consistent with LOF. We used the wt NOTCH1-knockout FADU HNSCC cell line with doxycycline-inducible cl-NOTCH1 expression as both a negative and positive control. As expected, cl-NOTCH1 staining was not detectable in knockout FADU in the absence of doxycycline, but strong nuclear staining was induced by doxycycline. In agreement with previous work,^13^ nuclear staining for cl-NOTCH1 was observed sporadically throughout the basal and immediate suprabasal layer in nonmalignant adjacent tonsil tissue from patient biopsies. However, staining for cl-NOTCH1 was not apparent in tumor regions from any of the study patients examined, consistent with NOTCH1 LOF mutations.

## Pharmacokinetics and Pharmacodynamics

**Table:** 

Variable	Day 1 (*n* = 6)	Day 8 (*n* = 6)	Day 22 (*n* = 5)	Day 43 (*n* = 4)	Day 64 (*n* = 5)
*C*_max_, ng/mL	574.5	574	691	525.5	546
Trough, ng/mL		62	101	11.6	139
*C*_max_ accumulation ratio		0.78	1.18	0.82	0.94
Trough accumulation ratio			1.11	0.94	0.63

### Result of Pharmacodynamic Analysis

Seven patients consented to PK studies, although patient 9 had only the day 64 PK draw owing to SARS-CoV-2 pandemic restrictions for research blood draws. The observed median *C*_max_ concentrations were similar across all 5 collection days: 574.5, 574, 691, 525.5, and 546 ng/mL on days 1, 8, 22, 43, and 64, respectively (*P* = .37). Median trough concentrations were 62, 101, 11.6, and 139 ng/mL on days 8, 22, 43, and 64, respectively, but the differences were not statistically significant (*P* = .66). Accumulation ratios for the *C*_max_ and trough values were calculated for each subsequent treatment day and compared with baseline (day 1 for *C*_max_ and day 8 for trough). Median values for the accumulation ratios were similar over time (*C*_max_: *P* = .72; trough: *P* = .65). We did not formally compare clinical response with PK values, but bimiralisib levels (*C*_max_ and trough) did not appear to be any higher in patients with a response (patients 4 and 9) or prolonged stable disease (patients 1 and 2) than in those with progression (patients 5 and 6).

## Assessment, Analysis, and Discussion

**Table:** 

Completion	Study completed
Investigator’s Assessment	Active and should be pursued further

Head and neck squamous cell carcinoma (HNSCC) is common and lethal despite recent advances with immunotherapy.^17-20^ Effective targeted therapies are still needed. A major challenge to personalized treatment for HNSCC based on genomic profiling is that most alterations are in tumor suppressors, including NOTCH1, which is mutated in ~20% of patients.^2,21-24^ The pattern of NOTCH1 mutations in HNSCC is consistent with LOF and supports the role of NOTCH1 as a tumor suppressor.^2^ There are no molecularly-targeted therapies approved for NOTCH1 mutant HNSCC, representing a significant unmet clinical need.

The PI3K/AKT/mTOR signaling pathway is one of the most frequently dysregulated pathways in cancer, including HNSCC.^15,25,26^ Only a few drugs that target the PI3K/AKT/mTOR pathway have been approved for clinical use^15^ and none for HNSCC. One reason for this lack of progress is a lack of any biomarker that predicts PI3K/AKT/mTOR pathway inhibitor activity, with the exception of the PIK3CA mutation in breast cancer.^27^ In other tumor types, PI3K/AKT/mTOR inhibitors have had limited clinical success even in the context of PIK3CA mutations.^15,28^

To address this gap, we have shown that HNSCC cell lines with NOTCH1 LOF mutations exhibit greater sensitivity to PI3K/mTOR inhibitors than their wild-type counterparts.^29^ PI3K/mTOR inhibition induced apoptosis in HNSCC cell lines harboring NOTCH1 LOF mutations. In contrast, HNSCC cell lines with PIK3CA mutations underwent cell cycle arrest,^30^ but not apoptosis, when treated with PI3K/mTOR inhibitors. In addition, PI3K/mTOR inhibitors reduced tumor growth in xenograft NOTCH1-mutant HNSCC models. In a phase I basket study of single-agent bimiralisib, one patient with NOTCH1-mutant HNSCC was treated, and this heavily pretreated patient experienced a partial response in thoracic metastases lasting for 9 months.^31^

Bimiralisib is a pan-class I PI3K/mTOR antagonist that potently inhibits PI3Kα and mTOR, with less potency against PI3Kβ. Bimiralisib does not significantly inhibit other protein kinases in biochemical assays^32^ and has a toxicity profile similar to other PI3K inhibitors.^8,11^ Pharmacodynamic data showed marked decreases of pathway targets in tumor tissue at therapeutic doses.^11^ Clinical responses and stable disease have been observed with bimiralisib.^10,11^

In patients with R/M HNSCC who had disease progression during cisplatin-based chemotherapy and immunotherapy, bimiralisib had an objective response rate of 17%, with a median OS and PFS of 7 and 5 months respectively. Although this trial was small, outcomes were better than the standard of care, which has an objective response rate of 5.8%, a median OS of 5.1 months, and PFS of 2.7 months (ie, the standard-of-care arm of reference^19^). However, the standard-of-care values do fall well within our 95% CIs.

The most significant limitation of the current study was that the SARS-CoV-2 pandemic adversely affected accrual, limited research blood collections, and led to sponsor insolvency and early termination. Additionally, patients with R/M HNSCC generally have high morbidity and mortality, as was evident not only in our bimiralisib-treated patients but also by two deaths in patients with a performance status < 2 who died from disease progression after consent but prior to study treatment. Collectively, these limitations led to a small number of evaluable patients.

The bimiralisib PK values in the current study are consistent with other published studies, which showed that the *C*_max_ value following administration of a single 120-mg dose was 876 ± 694 ng/mL.^8-11,31^ Given that the *C*_max_ concentrations proportionally increased with increasing doses up to 140 mg,^31^ we can compare PK data using the 150-mg dose in previous studies with PK data using the 140-mg dose in the current study. Wicki et al reported that the highest *C*_max_ was 998 ng/mL following a dose of 150 mg,^11^ which is similar to our maximum *C*_max_ value of 970 ng/mL, and the median *C*_max_ value did not differ significantly among treatment days tested. These *C*_max_ values are well above levels needs to inhibit PI3K and kill NOTCH1 mutant HNSCC cells.^29^ In our study, the intermittent dosing regimen allowed for partial clearance of the drug, and thus *C*_max_ accumulation was not observed. With a terminal half-life of 40 h, there were detectable trough concentrations throughout the treatment cycle in all patients; these trough concentrations reached a maximal value on day 64 but did not change significantly from day 8. A caveat of our PK data is that the *C*_max_ levels in our cohort were determined using a single time point at 1 hr after the oral dose and thus may not be reflective of the true *C*_max_ concentrations. Although the maximum plasma concentration in most humans occurs at 1-2 h after a dose, it can occur as late as 24 h after oral administration.^11^ However, our study design minimized PK variability driven by variable absorption depending on gastric pH and variable elimination via CYP1A2; concomitant drugs that affect gastric pH and CYP1A2 were not allowed.

Molecular testing of plasma-derived ctDNA can be used to detect the underlying tumor molecular profile and monitor its dynamic changes over time.^33^ In our study, we were able to detect NOTCH1 mutations in ctDNA collected at baseline in 83% of patients, which is consistent with previously published data for targeted next-generation sequencing.^34^ Dynamic changes in the ctDNA quantity during treatment were consistent with the clinical course. In addition, ctDNA samples collected at progression showed new emerging molecular alterations in major cancer genes such as PIK3CA, BRAF, TP53, and others; however, a small sample size precludes any definite conclusions.

The current study is the first clinical trial to test the therapeutic vulnerability of NOTCH1-mutant HNSCC to any class of drugs. Although the trial was small, the responses and prolonged stable disease are promising in a group of HNSCC patients with a very poor prognosis. These results, if confirmed in a larger trial, may inform the development of the first targeted therapy for NOTCH1 mutant HNSCC. Because NOTCH1 LOF mutations are common in other squamous cell carcinomas, including those of the skin, esophagus, and lung; our findings may translate beyond HNSCC.^35-38^

## Data Availability

The data underlying this article are available in the article and in its online supplementary material.
